# Prevalence and correlates of overweight and abdominal adiposity amongst adults residing in Madeira Autonomous Region: a cross-sectional, population-based study

**DOI:** 10.1097/j.pbj.0000000000000067

**Published:** 2020-07-17

**Authors:** Liliane P. da Costa, Eva S.A. Henriques, Teresa P.E. Gouveia

**Affiliations:** a Nutrition and Dietetic Unit from the Endocrinology Service; b Investigation Unit Dra. Maria Isabel Mendonça, Hospital Dr. Nélio Mendonça, Serviço de Saúde da Região Autónoma da Madeira, E.P.E, Funchal, Portugal.

**Keywords:** abdominal adiposity, adults, overweight

## Abstract

**Background:**

Data on nutritional status and its risk factors amongst the adult population of the Madeira Autonomous Region (RAM) is scarce. This study aims to investigate the prevalence of, and risk factors associated with overweight and abdominal adiposity, assessed through measuring body mass index (BMI), waist circumference (WC), and waist-to-height ratio (WHtR) indexes.

**Methods:**

Cross-sectional study using a representative sample of 911 subjects (18–64 years) from the RAM Dietary Habits of Adult Population Study. Logistic regression models were conducted to investigate the association between body mass index, WC, and WHtR indexes, with sociodemographic and lifestyle characteristics.

**Results:**

The prevalence of overweight amongst adults was 60.0% [95% confidence interval (CI): 56.8–63.2]. The prevalence of abdominal adiposity, assessed by WC and WHtR indexes, was 62.6% (95% CI: 59.4–65.7) and 71.9% (95% CI: 69.0–74.8), respectively. In adjusted models, age and self-reported chronic diseases were associated with both overweight and abdominal adiposity. Women were less likely to be overweight [odds ratio (OR) = 0.7 (95% CI: 0.5–0.9); *P* = .012] but more likely to have increased WC [OR = 2.9 (95% CI: 2.1–4.0); *P* < .001], compared to men. Being married was positively associated to being overweight [OR = 1.5 (95% CI: 1.1–2.1); *P* = .013] and increased WC [OR = 1.8 (95% CI: 1.3–2.6); *P* < .001], but not with WHtR index. Education level was only associated with WHtR index. Inverse associations were found for each abdominal obesity indicators and smoking status.

**Conclusions:**

Overweight and abdominal adiposity should be considered 2 major public health problems, amongst adult population of the RAM. Older less educated adults, with smoking habits may be considered a target group for health promotion interventions.

## Introduction

The Madeira Autonomous Region (RAM) is a Portuguese archipelago with approximately 267,785 residents, distributed by the 2 main islands, Madeira and Porto Santo.^1^ Located in the southern part of Madeira Island, Funchal, the capital of the RAM, was considered one of the most densely populated areas in the entire European Community.^2^ Data on nutritional status and its risk factors in adult population of the RAM is scarce. In 1998 to 1999, the Regional Secretary of Social Affairs of the RAM conducted the first, and unique (until now), regional dietary habits study (EHA-RAM 1998–1999), which showed results meaning that, 58.8% of the population (18–74 years old) were overweight.^3^ The Autonomous Regions of Madeira and Azores were included for the first time in 2005 to 2006 in a national survey, which results revealed that the islands had, in average, a higher body mass index (BMI) value.^4^ Kowalkowska et al^5^ found higher prevalence of overweight (59.7%) and abdominal adiposity (62.3%) in the RAM, compared to other Portuguese regions. Despite differences in sample groups age and methodology, other countries, such Spain and Greece, showed the same pattern of a higher prevalence of general or central obesity amongst the inhabitants in the islands.^6^,^7^ Addressing nutritional status of the islands populations, and documenting its determinants, risk factors, and correlates, are important research evidence for regional public health interventions. Therefore, in the present study, we aim (1) to investigate the prevalence of overweight and abdominal adiposity and (2) and to identify its associated sociodemographic and lifestyle factors.

## Methods

Data used in this study come from the RAM Dietary Habits of Adult Population Study (EHA-RAM *Estudo dos Hábitos Alimentares da RAM*), a study conducted by the Nutrition and Dietetic Unit from the Endocrinology Service of the Health Service of the RAM, between November 2012 and March 2015. The study was approved by the RAM Health Service Ethics Committee (ref: 03/2012) and all the participants provided written informed consent. Briefly, the target population for the EHA-RAM included all inhabitants, aged 18 to 64 years, registered at the health care centers of the RAM on June 18, 2012. The sample of the study was based on the 2011 Census data, published by the INE (*Instituto Nacional de Estatística/ Portugal Bureau of Statistics*), for age and region of residence (municipality). Overall, the random sample of the EHA-RAM included a total of 1800 subjects and the interview completion rate was 53.8%. Of the total surveys conducted, 36 were excluded due to invalid answers to key questions. The final sample of the EHA-RAM comprised 933 adults, and was considered representative of the adult population living in the RAM.

Detailed information was obtained from a questionnaire administered face-to-face, by nutritionists and dietitians, on sociodemographic information, self-perceived health status, and lifestyle behaviors such as food intake and dietary habits, physical activity, and smoking habits. Anthropometric measures were also assessed according to standard protocols.^8^ For the present study, we restricted analysis to an EHA-RAM subsample, applying the following exclusion criteria: lacking data concerning weight and/or height (n = 5), those participants with a BMI <18.5 kg/m^2^ (n = 17), and incomplete data related to social variables (n = 17). As a result, the final sample size comprised 911 eligible participants (61.1% women). The present work was reported according to the STROBE checklist for observational studies in epidemiology, provided as Supplemental Digital Content (SDC, File 1).^9^

Anthropometric parameters assessed for the present study were: height, weight, and waist circumference (WC). Standing height was measured to the nearest 0.1 cm using a stadiometer. Weight was measured to the nearest 0.1 kg using a TANITA TBF-300 body composition analyzer (Tanita Corp, Tokyo, Japan). WC was measured with a flexible nonelastic tape. BMI was calculated based on height and weight data, and categorized according to WHO as preobesity (25.0–29.9 kg/m^2^) and obesity (≥30.0 kg/m^2^).^10^ In the present study the term overweight was defined as BMI ≥25 kg/m^2^, and included both preobesity and obesity categories. Abdominal obesity was defined by increased WC (≥80 cm in women and WC ≥94 cm in men), and increased waist-to-height ratio (WHtR; >0.5 cm, for both sexes).^11^,^12^,^13^ Being overweight and having abdominal adiposity are the main outcome variables of this work.

Independent variables included sociodemographic characteristics and lifestyle variables. Demographic characteristics included sex, age (<30; 30–49; >49 years), and marital status (married, not married). Marital status category included those who were married or in civil partnership. Education levels were categorized in low (from lack of schooling up to attained 4 years of schooling), basic (has attained at least 6 years of the basic education, but <11); secondary education (if has attended 11 or 12 year of school); and higher education (if included university or polytechnic education). Job status was divided into employed (employee or self-employed) and not employed (unemployed, pensioners, students, housewives, or other status). Place of residence included the 11 municipalities organized into 4 zones: Funchal; West zone (included the municipalities of Câmara de Lobos, Ribeira Brava, Ponta do Sol and Calheta); North zone (Porto Moniz, São Vicente, and Santana); and East zone (Machico, Santa Cruz, and Porto Santo).

Smoking status was divided into smoker (if smoked currently or occasionally) and non/former smoker (if has stopped smoking or never smoked). Physical activity was measured using the short version of the International Physical Activity Questionnaire in metabolic equivalent hours per day, and classified into 3 levels: low, moderate, and high.^14^ Frequency of eating out variable was achieved by the combination of 2 questions: “During the last month, how often did you eat at restaurants?” and “During the last month, how often did you eat at fast-food restaurants?.” The responses scale for each event was “never,” “1 to 3 times/month,” “1 time/week,” “2 to 4 times/week,” “5 to 6 times/week,” and “every day.” Based on the distribution of responses and on previous research, that indicated an increased health risk associated with a consumption of eating out more than once per week, these categories were collapsed to “≤1 to 3 times/month” and “≥1 time/week.”^15^,^16^,^17^ Health self-reported status was also included in the present study, and “yes” or “no” dummy variables were created for the presence of diabetes, hypertension, dyslipidemia, or cardiac disease.

Statistical analysis was performed using the Statistical Package for Social Sciences (SPSS version 25.0) software. For all variables, a descriptive analysis was obtained, and Chi-square tests were performed to compare 2 categorical variables. A *P* value <.05 was considered statistically significant. Multivariate analysis (logistic regression) was done using the Forward Wald method. This method determines the inclusion or exclusion of variables into the model, step by step, until only those variables which explained the outcome remain in the analysis. Three logistic regression models were created to investigate the association between potential associated risk factors and: being overweight (Model 1); having increased WC (Model 2); and having increased WHtR (Model 3). Results were presented as odds ratio (OR) with 95% confidence interval (CI) and respective *P* values.

## Results

Table 1 describes the baseline characteristics of the study population according to nutritional status. Amongst the adult population of the RAM, the prevalence of overweight was 60.0% (95% CI: 56.8–63.2). The prevalence of abdominal adiposity, assessed by WC and WHtR indexes, was 62.6% (95% CI: 59.4–65.7) and 71.9% (95% CI: 69.0–74.8), respectively (Table 1).

**Table 1:**
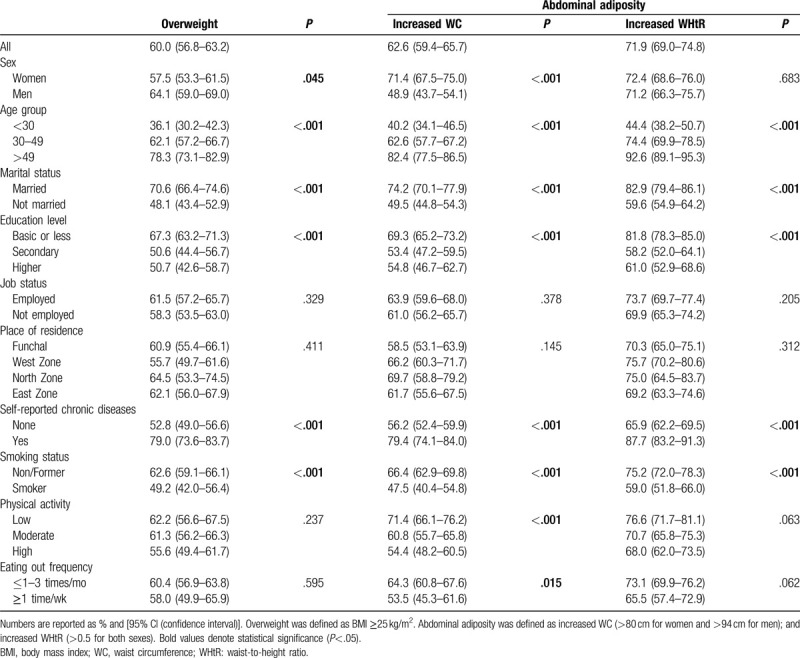
Baseline characteristics of study population

The proportion of overweight adults was higher amongst men than women [64.1% (95% CI: 59.0–69.0) vs 57.5% (95% CI: 53.3–61.5, *P* = .045]. Increased WC was more frequent in women than men [71.4% (95% CI: 67.5–75.0) vs 48.9% (95% CI: 43.7–54.1); *P* < .001]; however, no significant differences were found between sexes, for abdominal adiposity assessed by WHtR measure (*P* = .683).

The prevalence of abdominal adiposity, defined by WC index but not WHtR, was significantly higher in adults with low physical activity levels [71.4% (95% CI: 66.1–76.2); *P* < .001], compared to those with moderate or high activity; and in those who eat out less frequently [64.3% (95% CI: 60.8–67.6); *P* = .015], compared with adults that eat out ≥1 time per week (Table 1).

Table 2 shows results from 3 logistic regression models, to determine the independent risk factors for being overweight and having abdominal adiposity.

**Table 2:**
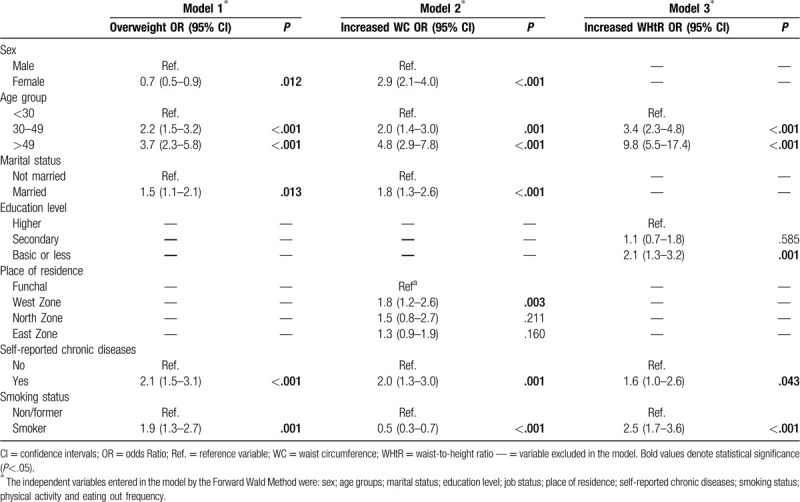
Adjusted odds ratio of being overweight and having central obesity

Based on the adjusted OR, results showed that women were less likely to be overweight [OR = 0.7 (95% CI: 0.5–0.9); *P* = .012] but more likely to have increased WC [OR = 2.9 (95% CI: 2.1–4.0); *P* < .001], compared to men. Being older and having a chronic disease were positively associated with both general and central obesity measures. Being married increased 1.5 times the odds of being overweight [OR = 1.5 (95% CI: 1.1–2.1); *P* = .013] and 1.8 the odds of having increased WC [OR = 1.8 (95% CI: 1.3–2.6); *P* < .001], but was not associated with increased WHtR. Education levels were only associated with WHtR index: less education level increased the odds of abdominal adiposity, compared to those with higher education [OR = 2.1 (95% CI: 1.3–3.2); *P* = .001]. Being a smoker increased 1.9 and 2.5 times the odds of being overweight (95% CI: 1.3–2.7; *P* = .001) and having an increased WHtR (95% CI: 1.7–3.6; *P* < .001), respectively. However, when abdominal adiposity was measured by WC, the risk is lower for smoker adults, compared to non/former smokers [OR = 0.5 (95% CI: 0.3–0.7); *P* < .001].

## Discussion

The present study provides up to date information on the prevalence and determinants of overweight and abdominal adiposity, amongst the adult population of the RAM. According to the EHA-RAM 2012–2015 database, approximately 60.0% of the adult population (18–64 years old) was considered overweight, and at least 3 in 5 adults had increased WC and WHtR indexes. Sex, education, marital status, and smoking habits were found to be associated, in the present study, with general or central adiposity. These results indicate a serious public health problem and should be compellingly tackled by appropriate health public policies within the RAM.

By the time the present survey was conducted, the European countries, including Portugal, were going through an economic and financial crisis. The RAM was the second national region with the greatest decrease in gross domestic product, with a huge impact in employment, which especially affected male young adults, with lower school education levels.^18^,^19^ Thereby, data obtained from the EHA-RAM 2012–2015, should be analyzed in the light of the economic and financial context of that time.

In the present study, amongst women and after adjustment for all variables, while the likelihood of being overweight was lower, the odds of having higher WC were higher. The higher prevalence of abdominal adiposity in women, compared to men, may be explained by genetic, hormonal, and physiological differences that influence body fat distribution.^20^

Notwithstanding, the indicator used for the screening of general and central obesity, the odds of being overweight and having abdominal adiposity were high in less educated and married adults. Our findings are in line with results from national works and from other European countries. The Kowakowska et al's^5^ work found an inverse relationship between education level (the highest educated ones as reference) and general [OR = 8.39 (95% CI: 5.13–13.71), *P* < .0001 for fourth completed grade adults] and abdominal adiposity [OR = 6.87 (95% CI: 4.23–11.15), *P* < .0001 for fourth completed grade adults]. In the same study, compared with married adults, and after adjustment for confounders, single subjects were negatively associated with increased WHtR [OR = 0.72 (95% CI: .54–0.97; *P* < .05)]. Results from the First Portuguese Health Examination Survey (INSEF 2015), that also included the RAM, concluded that age group (55–64 years old), educational level (no schooling/first cycle) and marital status (married) were the main factors associated positively with overweight.^21^ A representative cross-sectional study conducted in 2010 in 16 European countries (Portugal not included), on a total of 14,685 adults (aged ≥18 years), showed that prevalence of obesity increased with age [compared with <25 years old, *P* for trend <.001: OR = 3.82 (95% CI: 32.93–4.99) for 25–44 years; OR = 8.89 (95% CI: 6.83–11.56) for 45–64; and OR = 9.37 (95% CI: 7.12–12.33) for ≥65 years] and decreased with level of education [compared with low education, *P* for trend <.001: OR = 0.67 (95% CI: 0.59–0.76) for intermediate; and OR = 0.57 (95% CI: 0.49–0.67) for high education].^22^

Findings from our study are also in line with changes arising from the nutritional transition worldwide.^23^ According to the obesity transition theory, there has been a change in sociodemographic pattern of obesity, in the past 4 decades. Initially, by 1975, the prevalence of obesity did not exceeded 20%, and was distinctly higher among women and those with higher socioeconomic status, usually measured by school education degree and income. Over the years, the world assisted to a narrowing of the gender gap and a reversal of the socioeconomic differences.

After adjustment, in our study, age and self-reported chronic diseases were positively associated with both overweight and abdominal adiposity (increased WC and WHtR). These are the expected results, because fat mass increases with age and is a risk factor for other chronic diseases.^24^

Depending on which measurement was used, direction of association was found to be different for smoking habits. After adjustment, and compared with non/former smokers, being a smoker increases the odds of being overweight and having a higher WHtR, but an inverse relationship was found for WC. Controversial results have been observed in the literature. Previous studies showed that although smokers have lower mean BMI compared with nonsmokers, they have a more metabolically adverse fat distribution profile, with higher central adiposity.^25^ The population-based health examination survey in Finland, in 2007, found that, in overweight women, being a smoker elevates the WC, after adjustment for age, BMI, alcohol consumption, and physical activity index [β = 2.67 (95% CI: 1.85–3.50), *P* = 2.2 × 10^–10^ for occasionally and light/moderate daily smokers; and β = 4.48 (95% CI: 2.93–6.03), *P* = 1.5 × 10^–8^ for heavy daily smokers].^26^ More recently, genetic studies highlight the positive influence of smoking in genetic susceptibility to overall adiposity, but suggest that it may attenuate genetic effects on body fat distribution.^27^ At the same time, obesity may modify smoking behavior, gene expression, and the toxic effects of tobacco.^28^,^29^ Several biological mechanisms may explain the interaction between smoking and abdominal adiposity, such as the dysregulation of the hypothalamic-pituitary-adrenal axis, the action on regulation of the sex hormones and the ability to increase insulin resistance.^26^ In this study, BMI status was not used as a control variable, and there some body of evidence that the association patterns of regular smoking with central obesity is dependent on BMI adjustment.^26^,^30^ Despite disparities in WC and WHtR correlates, smoking habits and obesity are 2 of the major risk factors for noncommunicable diseases worldwide, and its reduction is an important way to control these diseases.^31^

Physical activity and the frequency in eating out were not found to be associated with either overweight or any measure of abdominal obesity, in the present study. Physical activity is a key determinant of energy expenditure and an important lifestyle behavior for controlling body composition.^32^ Eating out of home frequently, in classic or fast-food restaurants, is often associated with a poor quality of the diet, such as higher energy and fat intake, and consequently with increased risk of being overweight.^33^,^34^ It is possible that, because the financial and economic crises context, some people did change their habits although this had not yet reflected in an improvement of body composition. Indeed, the economic crises may have brought changes in the daily lives of families, for example, taking a lunch box to work and reducing gym expenses.

The present study has some limitations. First, the cross-sectional design provides evidence for associations but not causal relationships. Second, the study did not assess other important factors that may influence the outcomes, namely dietary habits and food intake. Other limitation is that the questionnaire administered was not validated in the study population. Finally, our analysis was not stratifying by sex, but its confounding effect was controlled and also minimized, by the use of sex-specific cutoff points for anthropometric measures predefined, when available. This study contains several important strengths. One of the major strength is that data were provided by the second regional survey of its kind, and enrolled a large number of subjects, essential for future comparisons with other regional and national studies. In addition, all anthropometric measures were assessed by trained personnel, resulting in more accurate values than self-reports. Another important strength is using both WC and WHtR indexes to assess abdominal adiposity, especially because the direction of the associations was not always consistent across the 2 measures. Therefore, further research on improving body weight–related indicators of adiposity is necessary.

In conclusion, overweight and abdominal adiposity should be considered 2 major public health problems, amongst adult population of RAM. Around 60.0% of the adult population (18–64 years old) was considered overweight, and at least 3 in 5 adults had abdominal adiposity. Regardless of different measures to assess body composition, significantly increased odds of overweight/ abdominal adiposity were observed amongst older and married adults, with low education level. Smoker adults and those with chronic conditions should also be considered a target group for health promotion interventions.

## Acknowledgments

Assistance with the study: Special thanks to the Training and Research Service of the Health Service of the Autonomous Region of Madeira for their contribution to the statistical analysis.

Financial support and sponsorship: none.

Presentation: none.

Author Contributions: All authors made substantial contributions to study conception, analysis, and the interpretation of data. L.P.C. was involved in conception and drafting the manuscript. E.S.A.H. contributed to the statistical analysis and interpretation of data. T.P.E.G. was involved in critically revising the important intellectual content. All authors read and approved the final version of the manuscript.

## Conflicts of interest

The authors declare no competing interests.
